# Functional Reaction Times of a Simulated Blocking Test among Para Taekwondo Athletes

**DOI:** 10.3390/healthcare10071231

**Published:** 2022-07-01

**Authors:** David Michael O’Sullivan, Hee Seong Jeong, Hyung Jin Won

**Affiliations:** 1 Division of Sport Science, Pusan National University, Busan 46241, Korea; davidosullivan@pusan.ac.kr; 2 Department of Sports and Health Management, Mokwon University, Daejeon 35349, Korea; 3 International Olympic Committee Research Centre Korea, Seoul 03722, Korea; 4 College of Do-ing, Tongmyong University, Busan 48520, Korea; whj945@hanmail.net

**Keywords:** classification, Paralympics, impaired, sport for all

## Abstract

Para taekwondo is a combat sport martial art that requires fast reaction times for successful defense during fighting. The current classification system is based on the function and the effective length of the upper limbs, which affects the athlete’s ability to block. Therefore, this study aimed to investigate the differences between the different classes in the athlete’s ability to block and move around the ring. A total of 87 Para taekwondo (K42, K43, and K44) athletes’ data were analyzed. Each participant engaged in the blocking reaction time test in a fighting stance with the left leg in front, the right leg in front, and the legs parallel in random order. A foot-stepping reaction test was performed to anlyze how the athletes moved in various directions. The results indicated no significant differences between the classes for the lower body foot stepping reaction times and the upper body blocking performance times. The stimulated blocking time of the Para taekwondo athletes ranged from 0.79 to 0.97 s Furthermore, the neurological group (0.86 ± 0.09 s) was significantly slower than the amputation/dysmelia group (0.81 ± 0.07 s). We thus concluded that the neurologically impaired athletes were disadvantaged and therefore belonged in a separate class.

## 1. Introduction

In 2015, Para taekwondo gained official recognition for inclusion in the 2020 Tokyo Paralympic Games [1]. Para taekwondo is different from taekwondo, the national sport of Korea, as the athletes in Para taekwondo are classified into similar groups according to their physical, visual, and intellectual impairments [2]. Additionally, the legal targets and the points allocation for techniques are different; Para taekwondo does not allow any head kicks (World Taekwondo rules and regulations) [3]. Since the first World Para Taekwondo Championships held in Baku, Azerbaijan, in 2009 [1], with just 38 competitors representing 19 countries, there has been an increase in the number of participating countries and competitors, with 400 competitors representing 69 countries in the eighth World Para Taekwondo Championships in 2019 [4]. Male and female athletes represented Kyorugi 41 (K41), and the Kyorugi 43 (K43) and Kyorugi 44 (K44) classes were consolidated [5].

Currently, there are two modalities of competition: Poomsae (the Korean word for forms) and Kyorugi (the Korean word for sparring). For Kyorugi, athletes with physical impairments, (i.e., neurological impairments, limb length differences, impaired range of motion, and amputations), compete in four classified groups—K41, K42, K43, and K44—with K41 being the group of athletes with the most severe impairments [3,6]. For Poomsae, athletes with physical, intellectual, and visual impairments are allowed to compete in varying classes from P31 to P34 [3,6]. As the athletes participating in Para taekwondo competitions have a large range of physical, visual, and intellectual impairments, classification is difficult. It is also challenging for athletes, coaches, and organizing committees participating in Para taekwondo to manage the competition events due to the difficulty of classifying athletes into their appropriate classes. As a result of the wide range of impairments in each class, Para taekwondo athletes are more likely to be exposed to a higher risk of injury [7]. Therefore, studies are needed to develop current classification systems based on the latest medical and scientific standards in order to prevent injuries, improve athletes’ performance, and ensure fair competition [8,9,10].

For this study, we focused on the K-class athletes, i.e., the Kyorugi-participating athletes [8]. Until 2021, the four K-classes were mainly split into groups according to the athletes’ potential to block actively and passively [11]. Therefore, the main objective of this study was to investigate the differences in active blocking and footwork between the K42, K43, and K44 classes to learn more about the K-classes, which will help improve the current classification criteria. A secondary aim of the study was to examine the outcomes of consolidated Kyorugi matches (with the mixing of K-classes due to the lack of competitors) to see the extent to which the classification affects the outcome of winning or losing. We aimed to investigate the way in which the upper body impairment-based classes K42, K43, and K44 affect the reaction time to simulate blocking. We also aimed to examine the effect that the differences between the type of impairments, i.e., neurological impairment or amputation, had on the reaction time to simulate blocking. Lastly, we aimed to examine a performance-based outcome of the probability of a lower class winning if the class was consolidated with a more difficult or less impaired class.

## 2. Materials and Methods

### 2.1. Participants

Prior to any of the athletes participating in this study, World Taekwondo (WT) approved the consent form that was formalized and reviewed by WT international lawyers and the ethical committee of Yonsei University (IRB no. 7001988-201708-HR-245-04) in accordance with the Declaration of Helsinki. Participants were asked to voluntarily participate in this study after a verbal explanation and signing the consent form. A total of 92 athletes ((weight: 59.9 ± 13.5 kg (males), 58.2 ± 14.6 kg (females); height: 173.7 ± 7.8 cm (males), 160.5 ± 6.9 cm; training per week: 4.4 ± 1.8 times/week (male and female)) participated prior to two large international Para Taekwondo Official WT competition events. The first round of data collection was held in Amman, Jordan, and the data collection occurred 2 days prior to the competition (17 July 2019) with 29 athletes participating. The second round of data collection was held in Bari, Italy, two days prior to the competition (29–30 October 2019) with 63 athletes participating.

A total of 92 athletes participated in this study and had their upper body blocking reaction time recorded for three trials each, totaling 276 trials. However, as five participants turned out to be coaches and not para-athletes, we had to remove their data; this left us with the data of a total of 87 para-athletes to be analyzed. Due to convenience sampling at prior competitions and the fact that only the athletes who volunteered could participate, there was an odd number of athletes in the three classes tested. There were 6 K42 athletes, 18 K43 athletes, and 63 K44 athletes, which is the ratio of athletes attending the WT competitions.

### 2.2. Equipment

**Figure 1:**
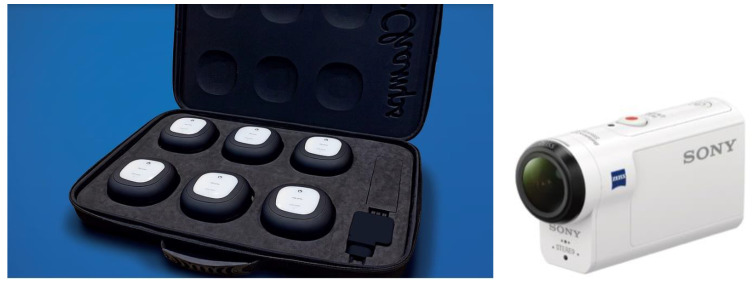
(**left**) ROXs Pro System© with the charging and storage case for mobility. A tablet PC or mobile phone (IOS or Android) is needed to control the ROXs Pro System© settings and show the output data. (**right**) Sony Action camera to record movements.

ROXs Pro© ((A-champs, Madrid, Spain) was used for measuring and recording the individual’s reaction time to block each light (https://www.a-champs.com/ (accessed on 27 June 2020))). Each of the participants’ data was saved automatically on the ROXs Pro© company-provided cloud, which was password protected. All of the athletes’ movement patterns were recorded using Sony Action Camera at 120 frames per second (Figure 1).

**Figure 2:**
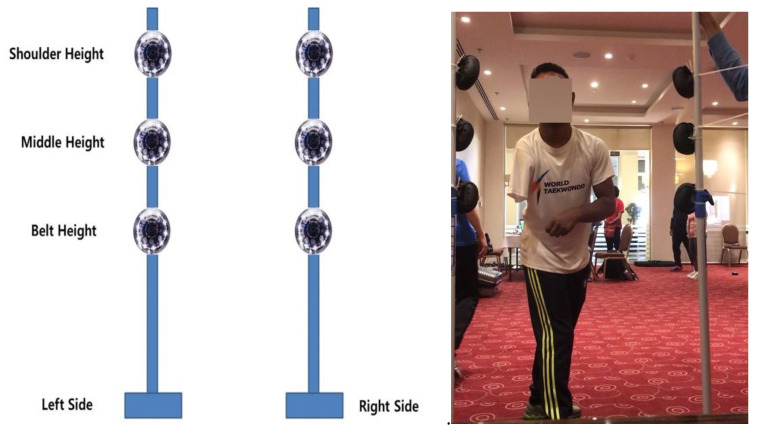
Front view of ROXs Pro sensors set up individually for each athlete’s height; left (schematic), right (data collection).

**Figure 3:**
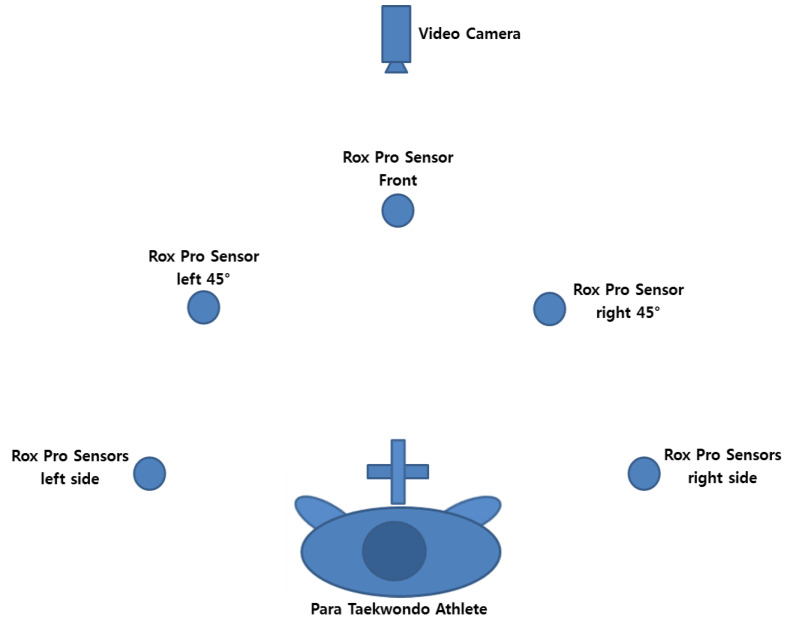
Overview of taekwondo stepping reaction test.

The six ROXs Pro system pods were set up on two stands that were weighed down and placed on the left and right sides of the athlete. The pillars were placed 90 cm apart with the athlete point positioned 30 cm from the center of the line between the pillars. The belt, middle, and shoulder height were set with a spacing of 20 cm between belt height and middle height and 20 cm between middle height and shoulder height, and the height was adjusted according to the athlete’s hip. The belt (waist) height was taken from the average 50th percentile 40-year-old male data. The waist-to-shoulder distance was also taken from the average 50th percentile 40-year-old male data, which was 65.4 cm. When sparring in a fighting stance, the athlete’s legs are bent, so their height is slightly reduced. Therefore, for convenience and standardization among each of the athletes, we selected a belt height of 100 cm, a middle height of 130 cm, and a shoulder height of 160 cm. Each of the test pods was numbered from 1 to 6: the top left was 1, the middle left was 2, the bottom left was 3, the top right was 4, the middle right was 5, and the bottom right was 6. The ROXs Pods were placed as shown in Figure 2 and Figure 3.

### 2.3. Testing Procedure

1. All athletes from K42, K43, and K44 attending the event were asked to participate in the study.
2. After a verbal explanation of the test procedure, the athlete signed the written consent form to participate in the test.
3. Prior to warming up, the athletes’ WT-assigned GAL number was recorded so that the researchers would have access to the athletes’ classification data, which included any information about their impairments.
4. Each athlete had to perform a series of 30 blocks and hit the ROXs Pro Pods, each randomly selected, for the three heights and both the left and right side, 15 times on each side.
5. The test began with the right leg forward in a fighting stance, then the left leg forward in a fighting stance, and the legs parallel 30 cm in front of the targets. Thus, all athletes performed a total of 90 blocks at random for the three fighting stances.
6. The foot movement performance timing involved stepping on the ROXs Pro sensors; the participant had to stand with the feet shoulder-width apart with a distance of 1 m to each of the five sensors placed on the ground. The distance of 1 m was selected so as to force the athlete to have to step instead of reach.
7. Reaction times for the upper body blocking time and the lower body stepping agility and movement times were saved automatically by the ROXs Pro training system and were downloaded in Microsoft Excel format for analysis.

### 2.4. Statistical Analysis

Matlab (version R-2019) was used to produce the descriptive statistics (mean ± standard deviation), repeated-measures ANOVA, independent *t*-test, and Chi-squared tests. Repeated-measures ANOVA was applied to investigate the differences between the K classes. The independent *t*-test was applied to investigate the difference between the neurological impairment and the amputation/dysmelia group. Chi-squared tests were applied to verify the probability of the winning-to-losing distribution for the consolidated matches. The level of significance was set at *p* < 0.05 for all statistical tests. Cohen’s d effects size was calculated for all variables that had significant differences and interpreted as follows: less than 0.2 was small, 0.5 was medium, and 0.8 was large [12]. As a result of using a power of 0.80, an effect size of 0.40, two tails, and a significance level of 0.05 using G∙POWER (G-power program 3.1.9.7, Heinrich-Heine-University, Düsseldorf, Germany) to determine the post hoc statistical power, at least 52 study participants were required [13], and this study was conducted with a total of 92 subjects.

## 3. Results

A total of 92 athletes participated in this study, and Table 1 shows the descriptive statistics of the K42, K43, and K44 classes for the upper body blocking reaction time and the lower body stepping reaction time. The K44 athletes were divided into two groups: neurologically impaired and athletes with dysmelia or amputation. Table 2 shows the descriptive statistics of the neurologically impaired and athletes with dysmelia or amputation.

### 3.1. Blocking Reaction and Lower Body Stepping Times

According to the repeated measures ANOVA analysis ((F (2,259) = 0.52, *p* = 0.59, effect size (partial eta squared) = 0.003, statistical post hoc power of 85.1%)), there were no statistical differences between the K classes. Likewise, there was no statistically significant difference ((F (2,259) = 1.18, *p* = 0.2472)) between the K classes for the lower body stepping reaction time. However, within the K44 class, there was a significant difference between the neurological and amputation/dysmelia group ((t(175) = 24.6, *p* < 0.001)) with a large Cohen’s d effect size of 0.65 and statistical post hoc power of 79.3%.

### 3.2. Consolidation Match Results

A total of 97 matches at WT sanctioned competitions, which had to be consolidated with another class from the year 2015 to 2020, were included in the analysis, as shown in Table 3. The results of these matches were analyzed using the Chi-squared test to verify the probability of the winning-to-losing distribution. In the consolidated matches between K42 and K44, in a total of 16 matches, K42 had 6 wins and 10 losses. The Chi-squared statistical method showed a probability of 32% of the data being different. Therefore, we concluded that K42 and K44 athletes competing together may be fair. Between the K42 and K43 matches, in a total of 21 matches, K42 had 9 wins and 12 losses. Similarly, the Chi-squared statistical method showed a probability of 42% of the data being different. Therefore, we concluded that K42 and K43 athletes competing together may be fair. However, in the consolidated matches between K43 and K44, in a total of 60 matches, K43 had 22 wins and 38 losses. The Chi-squared statistical method showed a probability of 96% of the data being different. Therefore, we concluded that K43 and K44 athletes competing together may not be fair.

## 4. Discussion

This study aimed to test the reaction times of simulated blocking and footwork of K42, K43, and K44 classes to investigate the differences in the ability of Para taekwondo athletes to block and move during competition. The main results showed that there were no significant differences between the K42, K43, and K44 classes for both the lower body foot stepping reaction time and the upper body blocking performance times. As the K classes were separated by the athlete’s upper body impairments, we were surprised that there were no significant differences for the upper body simulated blocking reaction times. However, as expected, there was no significant difference between the K classes for the foot stepping reaction times. The simulated blocking time for Para taekwondo athletes ranged from 0.79 s to 0.97 s. Unfortunately, there are no other known studies that have recorded the same blocking reaction times. However, these results are similar to the performance times recorded for the roundhouse kick in elite taekwondo athletes of 0.80 ± 0.01 s [14], which cannot be used in direct comparison.

The results also suggested the reaction times of the neurological group (0.86 ± 0.09 s) were significantly slower than those of the amputation/dysmelia group (0.81 ± 0.07 s). These results are consistent with existing research showing that neurological disorders may slow voluntary and functional movement times [15,16]. These results are particularly important because they provide evidence that neurologically impaired athletes may be at a disadvantage in combat situations [17], as taekwondo [14] requires rapid reactions and performance times. Previous studies have identified various factors, such as age, gender, caffeine, and training time, that affect reaction and performance times in taekwondo [18,19]. A limitation that must be considered is that we were not able to control the athlete’s caffeine intake for the data collection due to the athletes having to compete within the next two days.

As Para taekwondo has a total of 32 classifications (four weight classes, two genders, and four K classes), it is difficult for each competition to fill each division with an appropriate number of competitors for competitions (WT rules) [3]. This lack of competitors in a specific division may force the competition’s organizing committee to consolidate athletes with a more severe classification into a less severe classification so that the athlete can participate (WT organizing committee rules and regulations) [20,21,22]. We analyzed the results of the 92 consolidated matches from the past five years (2015–2020) to see if we could estimate how fair this consolidation was. Our statistical analysis of the data showed that K42 and K44 athletes competing together may be fair, K42 and K43 athletes competing together may be fair, and K43 and K44 athletes competing together may not be fair. An important issue to note here is that the lack of consolidated matches between the more severe K classes (K42 and K43) meant that they had fewer competitors than the K44 class, which may negatively affect the data.

### Recommendations and Limitations

A limitation in the study arose due to our method of measuring the participant’s performance time, i.e., the time it takes for them to react from the stimulus light to the hitting of the sensor; we were not able to separate the athlete’s performance and reaction time. However, for the convenience of the readers, we used the wording of reaction time throughout the paper. Another limitation is that the IPC highly recommends against using performance-related factors to classify athletes. However, here we provide the performance-based variables to measure the effect that the impairment may have on the functional performance of blocking during competition. Another limitation is that because the K44 class has the largest number of athletes with the lowest severity of impairments, we observed a higher number of competitors in this class and a higher standard of the athletes due to the higher level of competition in the K44 class compared with athletes with more severe impairments (K42 and K43). Additionally, as K44 athletes have a higher chance of qualifying for the Paralympics, there tends to be more financial and logistical support for these athletes [8].

Due to the relatively low number of total elite Para taekwondo athletes worldwide, the statistical power of this study is limited. Future monitoring of the fairness of competition between the Para athletes should be continued as the sport develops. Such a low number of Para taekwondo athletes compounded by the travel restrictions due to COVID-19 hindered the opportunity to collect more data for further analysis prior to the national and international competitions. We strongly recommend continued monitoring of all competitions, especially in terms of injury surveillance, to ensure safe competition and to recruit additional athletes for functional testing. With the latest World Taekwondo injury surveillance and continued research, studies should be focused on injury surveillance to be able to help develop effective injury prevention programs. Another limitation of this study is the lack of comparative data due to the lack of combat sports that have been adapted to the Paralympic program.

## 5. Conclusions

This study showed that there were no significant differences in the blocking reaction times between the K42, K43, and K44 classes. However, it highlights the fact that athletes with neurological disorders are disadvantaged compared with athletes with amputation/dysmelia for functional blocking during competitions. Additionally, the results of the consolidated matches indicate the possible fairness of K42, K43, and K44 athletes competing together. In conclusion, athletes with neurological disorders should be studied further to ensure that they can participate in safe competitions.

## Figures and Tables

**Table 1:** Difference between the reaction times for the K42, K43, and K44 athletes.

Groups (Total 261 Trials)	Reaction Time (s)	
	Upper Body Blocking (s)	Lower Body Stepping (s)
K44 Group (*n* = 189)	0.81 ± 0.06	1.51 ± 0.29
K43 Group (*n* = 54)	0.98 ± 0.24	1.59 ± 0.34
K42 Group (*n* = 18)	0.79 ± 0.04	1.49 ± 0.24

**Table 2:** Difference between the neurological impairments and amputation/dysmelia reaction time.

Groups (Total Trials 176)	Reaction Time (s)	*p*-Value	Effect Size Cohen’s d
Neurological Group (*n* = 27)	0.86 ± 0.09	<0.001	0.65
Amputation/Dysmelia (*n* = 149)	0.81 ± 0.06		

**Table 3:** Results of Chi-squared analysis for the consolidated matches.

Consolidated Match Groups	Total Matches	Wins	Losses	Chi-Squared
K42 with K44	16	6	10	0.32
K42 and K43	21	9	12	0.42
K43 and K44	60	22	38	0.96

## Data Availability

Not applicable.
